# Synchronous multiple primary tumors in patients with malignant lymphoma: a retrospective study

**DOI:** 10.1186/s12885-022-09734-7

**Published:** 2022-06-11

**Authors:** Yu Yagi, Yusuke Kanemasa, Yuki Sasaki, An Ohigashi, Yuka Morita, Taichi Tamura, Shohei Nakamura, Akihiko Kageyama, Yasushi Omuro, Tatsu Shimoyama

**Affiliations:** 1 Department of Medical Oncology, Tokyo Metropolitan Cancer and Infectious Diseases Center, Komagome Hospital, 3-18-22 Hon-komagome, Bunkyo-ku, Tokyo, 113-8677 Japan; 2 Department of Clinical Research Support, Tokyo Metropolitan Cancer and Infectious Diseases Center, Komagome Hospital, Tokyo, Japan

**Keywords:** Synchronous multiple primary malignant tumors, Lymphoma, Solid tumor, Survival

## Abstract

**Background:**

Synchronous multiple primary malignant tumors (sMPMTs) are sometimes diagnosed in patients with malignant lymphoma. We herein investigated the prognostic impact of sMPMT in lymphoma patients and the optimal treatment strategy.

**Methods:**

Seventy-five patients with sMPMTs (5.8%) among 1285 patients with lymphoma newly diagnosed between August 2004 and April 2020 were enrolled.

**Results:**

In patients with indolent lymphoma, those with sMPMTs had a worse prognosis than those without sMPMTs (5-year overall survival [OS]: 73.4% and 87.8%, respectively; *P* = 0.047). Among those with high and low tumor burden, the cumulative rate of death due to solid tumors was significantly higher in patients with sMPMTs than those without sMPMTs (high tumor burden: 26.7% vs. 1.6%, *P* < 0.001; low tumor burden: 12.7% vs. 1.0%, *P* = 0.003). The presence of sMPMTs did not have a significant impact on outcomes in patients with diffuse large B-cell lymphoma (DLBCL) (5-year OS: 65.4% and 66.9%, respectively; *P* = 0.74; 5-year progression-free survival [PFS]: 65.5% and 59.9%, respectively; *P* = 0.65). However, the cumulative rate of death from solid tumor in patients with sMPMTs was significantly higher than in patients without sMPMTs (5-year cumulative rate: 7.4% and 2.1%, respectively; *P* = 0.004). The treatment sequence did not have a significant effect on outcomes or the relative dose intensity of chemotherapy.

**Conclusions:**

In patients with indolent lymphoma, those with sMPMTs had a significantly worse prognosis than those without sMPMTs, mainly because of high mortality due to solid tumors. The presence of sMPMTs was not a significant prognostic factor in patients with DLBCL. It is important to assess the status and need for early treatment of each type of malignancy in patients with sMPMTs.

## Background

Synchronous multiple primary malignant tumors (sMPMTs) are sometimes diagnosed during screening tests in patients with newly diagnosed malignant neoplasms. The frequency of MPMTs is reportedly in the range of 2–17% [1]. This may also be true for non-Hodgkin’s lymphoma, the diagnosis of which usually involves a systematic, whole-body examination, which may detect more sMPMTs [2]. Between 3.1 and 3.6% of patients with diffuse large B-cell lymphoma (DLBCL) reportedly had sMPMTs [3, 4].

The aim of lymphoma treatment depends on histology. Patients with many types of aggressive lymphoma, such as DLBCL, receive curative chemotherapy. These patients have a 5-year survival rate of about 80% [5, 6]. In contrast, patients with low-grade B-cell lymphoma, including follicular lymphoma and marginal zone lymphoma, receive palliative, rather than curative, chemotherapy. However, patients with low-grade lymphoma may enjoy longer survival; patients younger than 40 years with follicular lymphoma have a median overall survival (OS) of 24 years with a 10-year OS rate of 81% [7].

For the treatment of solid tumors, patients with localized disease generally receive local therapy, such as surgery or radiotherapy, with or without adjuvant therapy with curative intent. Patients with advanced disease who are unsuitable for curative therapy receive palliative chemotherapy. In patients with multiple, advanced solid tumors, chemotherapy for each tumor type may overlap. However, drugs that are central to the treatment of lymphoma, such as doxorubicin and bendamustine, are rarely used to treat solid tumors. Thus, treating sMPMTs in lymphomas or advanced solid tumors simultaneously with chemotherapy is difficult, and the order of their treatment needs to be decided. However, the optimal treatment strategy for sMPMTs has not been established because patients with this disease are usually excluded from clinical trials [8]. If the solid tumor is treated first, the treatment of the lymphoma will be delayed, possibly worsening the prognosis [9–11]. On the other hand, if the lymphoma is treated first and treatment of the solid tumor begins during the lymphoma treatment, interruption of chemotherapy can lead to low relative dose intensity and poor outcomes [12–14]. Of course, if treatment for the solid tumor is delayed, the tumor may progress. Physicians often face these dilemmas when treating sMPMTs.

In the present study, we retrospectively investigated the prognostic impact of sMPMT in patients with malignant lymphoma and assessed the outcomes of different treatment strategies.

## Methods

### Patients

The medical records of patients with lymphoma newly diagnosed between August 2004 and April 2020 in our department were reviewed. sMPMTs were defined as more than two malignancies detected within 6 months [15]. Lymphomas were pathologically diagnosed in accordance with the World Health Organization (WHO) classification [16, 17]. Clinical staging was performed using the Ann Arbor Classification. Performance status (PS) was evaluated using the Eastern Cooperative Oncology Group (ECOG) criteria. The International Prognostic Index (IPI) and National Comprehensive Cancer Network-International Prognostic Index (NCCN-IPI) scores were calculated based on age, serum lactate dehydrogenase (LDH), PS, Ann Arbor stage, and extranodal involvement at diagnosis [18, 19]. The tumor burden in patients with indolent lymphoma was determined on the basis of the Groupe d’Etude des Lymphomes Folliculaires (GELF) criteria. The clinical tumor response was assessed using computed tomography (CT) or positron emission tomography-CT (PET-CT) according to the International Workshop Criteria or Lugano Criteria [20, 21]. Solid tumors for which curative treatment, such as surgical resection and radiotherapy with or without chemotherapy, was indicated were defined as localized tumors. Tumors for which curative treatment was not indicated were classified as advanced tumors.

### Relative dose intensity

The standard R-CHOP regimen, consisting of rituximab (375 mg/m^2^ on day 1), cyclophosphamide (750 mg/m^2^ on day 2), doxorubicin (50 mg/m2 on day 2), vincristine (1.4 mg/m^2^, max 2 mg/body on day 2), and prednisone (100 mg/day on days 2–6), was administered every 3 weeks. The initial R-CHOP dose was often reduced based on previous reports [22, 23]. R-THP-COP included tetrahydropyranyl adriamycin (30 mg/m2 on day 2) instead of doxorubicin. Dose modifications and the timing of the start of subsequent cycles were decided at the physicians’ discretion. The delivered dose intensity was calculated as the total delivered dose divided by the total time until completion of the chemotherapy. The relative dose intensity (RDI) was calculated as the percentage of the delivered dose intensity divided by the standard intensity [24]. The RDI of R-CHOP and R-THP-COP was defined as the average relative dose of cyclophosphamide, doxorubicin, or tetrahydropyranyl adriamycin. To analyze the clinical impact of treatment intensity accurately, patients who received fewer than three courses of R-CHOP or R-THP-COP were excluded.

### Statistical analysis

OS was defined as the time from the date of diagnosis of the solid tumors or lymphoma, whichever was diagnosed first, and the last follow-up or death from any cause. Progression-free survival (PFS) was defined as the time from the date of diagnosis of the solid tumors or lymphoma to the last follow-up, documented progression, relapse, or death from any cause. OS and PFS were estimated using the Kaplan-Meier method and were compared using univariate analysis with the log-rank test. Cumulative incidence of death from lymphoma and death from solid tumor was evaluated using Gray’s method, with the risk of each considered a competing risk [25]. The differences in the characteristics of the two groups were assessed using Fisher’s exact test or Student’s *t*-test. Student’s *t*-test was also used to compare the RDI, and the Mann–Whitney *U* test was used to compare the interval in days between diagnosis and treatment. All *P* values were two-sided, and *P* = 0.05 or less was considered to indicate statistical significance. The statistical analysis of survival and cumulative incidence was performed using EZR software [26].

## Results

### Patient characteristics

Seventy-five of 1285 patients with lymphoma had sMPMTs. Table 1 shows the characteristics of these patients. The median follow-up time was 50.9 months (range: 1–136 months) and the median age at diagnosis was 70 years (range: 46–91 years). Thirty-six patients (48.0%) had aggressive lymphoma, and 39 (52.0%) had indolent lymphoma. With regard to solid tumors, 68 patients (90.7%) had localized tumors, and seven (9.3%) had advanced tumors.

**Table 1:** Patient characteristics

	Aggressive lymphoma (*n* = 36)	High tumor burden indolent lymphoma (*n* = 12)	Low tumor burden indolent lymphoma (*n*=27)	*P* value
Lymphoma subtype				-
DLBCL	28 (77.8%)	-	-	
AITL	3 (8.3%)	-	-	
PTCL-NOS	2 (5.6%)	-	-	
HL	2 (5.6%)	-	-	
ATLL	1 (2.8%)	-	-	
FL, grade1, 2, and 3A	-	6 (50.0%)	19 (70.4%)	
MZL	-	6 (50.0%)	7 (25.9%)	
CLL/SLL	-	0	1 (3.7%)	
sMPMTs				
Localized or advanced				0.63
Localized	33 (91.7%)	10 (83.3%)	25 (92.6%)	
Advanced	3 (8.3%)	2 (16.7%)	2 (7.4%)	
Cancer type				0.27
Gastric	13 (36.1%)	2 (16.7%)	9 (33.3%)	
Colorectal	8 (22.2%)	6 (50.0%)	7 (25.9%)	
Lung	4 (11.1%)	3 (25.0%)	1 (3.7%)	
Bladder	4 (11.1%)	0	1 (3.7%)	
Breast	1 (2.8%)	0	3 (11.1%)	
Kidney	3 (8.3%)	0	0	
Prostatic	1 (2.8%)	0	1 (3.7%)	
Endometrial	1 (2.8%)	0	0	
Esophageal	1 (2.8%)	0	0	
Ovarian	0	1 (8.3%)	0	
Paranasal	0	0	1 (3.7%)	
Pancreatic	0	0	1 (3.7%)	
Thyroid	0	0	1 (3.7%)	
Thymoma	0	0	1 (3.7%)	
Skin cancer	0	0	1 (3.7%)	

*DLBCL* diffuse large B-cell lymphoma, *AITL* angioimmunoblastic T-cell lymphoma, *PTCL-NOS* peripheral T-cell lymphoma, not otherwise specified, *HL* Hodgkin lymphoma, *ATLL* adult T-cell leukemia/lymphoma, *FL* follicular lymphoma, *MZL* marginal zone lymphoma, *CLL* chronic lymphocytic leukemia, *SLL* small lymphocytic lymphoma, *sMPMTs* synchronous multiple primary malignant tumors

### Indolent lymphoma

Among 502 patients with indolent lymphoma, 39 patients had sMPMTs (12 had a high tumor burden and 27 had a low tumor burden). The breakdown of the histology revealed 25 cases of follicular lymphoma (grade 1, 2, or 3A), 13 cases of marginal zone lymphoma, and one case of chronic lymphocytic leukemia/small lymphocytic lymphoma. Lymphoma or solid tumor was diagnosed first in 17 (43.6%) and 20 (51.3%) patients, respectively, while they were diagnosed simultaneously in two (5.1%) patients. Almost all patients with a high tumor burden received some anti-lymphoma treatment (rituximab-containing chemotherapy or radiation), but about 70% of the patients with a low tumor burden were only observed. Half the patients with a high tumor burden and almost all the patients with a low tumor burden received treatment for the solid tumor first (Table 2).

**Table 2:** Characteristics of patients with indolent lymphoma

	High tumor burden indolent lymphoma (*n* = 12)	Low tumor burden indolent lymphoma (*n*=27)	*P* value
Initial treatment for lymhpoma			<0.001
Rituximab	2 (16.7%)	0	
Rituximab+chemothrapy	7 (58.3%)	3 (11.1%)	
Radiotherapy	2 (16.7%)	2 (7.4%)	
Eradication of *H.pylori*	0	3 (11.1%)	
Watch and wait	1 (8.3%)	19 (70.4%)	
Treatment for solid tumor			0.71
Surgery ± chemotherapy	9 (75.0%)	13 (48.1%)	
Endocope	2 (16.7%)	8 (29.6%)	
Radiotherapy ± chemotherapy	1 (8.3%)	3 (11.1%)	
Chemotherapy	0	2 (7.4%)	
Cystoscope	0	1 (3.7%)	
Treatment sequence			0.002
Lymphoma first	6 (50.0%)	1 (3.7%)	
Solid tumor first	6 (50.0%)	26 (96.3%)	

**Fig. 1:**
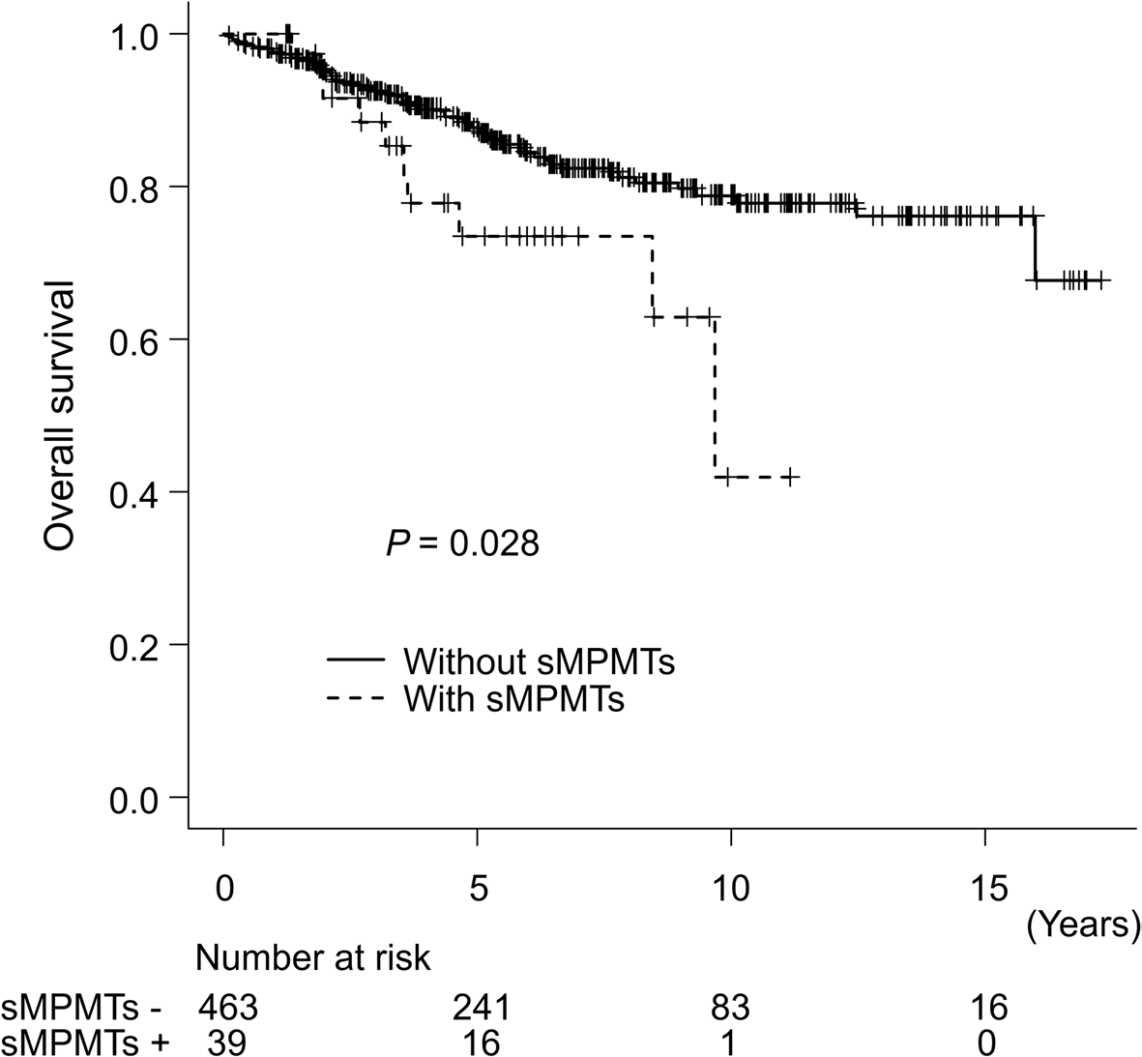
Kaplan-Meier curves of overall survival stratified by the presence of sMPMTs in patients with indolent lymphoma

**Fig. 2:**
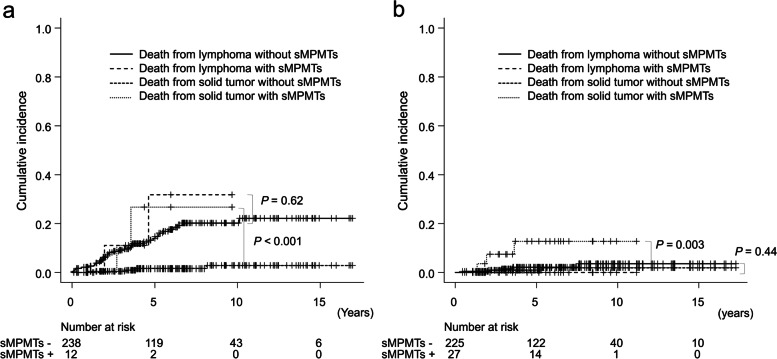
Cumulative incidence of death due to lymphoma or solid tumor stratified by the presence of sMPMTs in patients with high tumor burden indolent lymphoma (**a**) and in those with low tumor burden indolent lymphoma (**b**)

Five-year OS was significantly worse in patients with sMPMTs than those without sMPMTs (73.4% and 87.8%, respectively; *P* = 0.028) (Fig. 1). In patients with high tumor burden, although there was no significant difference in the cumulative rate of death due to lymphoma at 5 years between patients with and without sMPMTs (31.9% vs. 13.9%, *P* = 0.62), the 5-year cumulative rate of death due to a solid tumor in patients with sMPMTs was significantly higher than those without sMPMTs (26.7% and 1.6%, respectively; *P* < 0.001) (Fig. 2a). Similarly, in patients with low tumor burden, the 5-year cumulative rate of death due to a solid tumor in patients with sMPMTs was significantly higher than those without sMPMTs (12.7% and 1.0%, respectively; *P* = 0.003), and the 5-year cumulative rates of death due to lymphoma in patients with without sMPMTs were nearly identical, but very low compared to those in patients with high tumor burden (0% and 2.2%, respectively; *P* = 0.44) (Fig. 2b).

### DLBCL

Twenty-eight patients had DLBCL with sMPMTs. Table 3 compares the characteristics of the patients with and without sMPMTs. Lymphoma or solid tumor was diagnosed first in 21 (75.0%) and six (21.4%) patients, respectively, while they were diagnosed simultaneously in one (3.6%) patient. Curative therapy for solid tumors was performed prior to, during, and after treatment for lymphoma in nine (32.1%), seven (25.0%), and four (14.3 %) patients, respectively. Nine patients received surgery, 8 patients received endoscopic treatment, and 3 patients received cystoscopic resection for bladder cancer. Eight (28.6%) patients did not receive curative therapy for their solid tumor.

**Table 3:** Characteristics of patients with DLBCL

	DLBCL with sMPMTs (*n*=28)	DLBCL without sMPMTs (*n*=671)	*P* value
Age (years)			
Range, median	49–91, 75.5	27–97, 70	
Age > 60	24 (85.7%)	511 (76.2%)	0.36
Sex (male)	21 (75.0%)	361 (53.8%)	0.032
B-symptoms (+)	4 (14.3%)	174 (25.9%)	0.19
ECOG-PS (≥ 2)	2 (7.1%)	193 (28.8%)	0.009
LDH (> ULN)	15 (53.6%)	383 (57.1%)	0.70
Ann Arbor stage (3/4)	11 (39.3%)	370 (55.1%)	0.12
Extranodal involvement (≥ 2)	5 (17.9%)	185 (27.6%)	0.39
NCCN-IPI (HI/H)	15 (53.6%)	397 (59.2%)	0.56

*DLBCL* diffuse large B-cell lymphoma, *sMPMTs* synchronous multiple primary malignant tumors, *ECOG-PS* Eastern Cooperative Oncology Group performance status, *LDH* lactate dehydrogenase, *ULN* upper limit of normal, *NCCN-IPI* National Comprehensive Cancer Network-International Prognostic Index

**Fig. 3:**
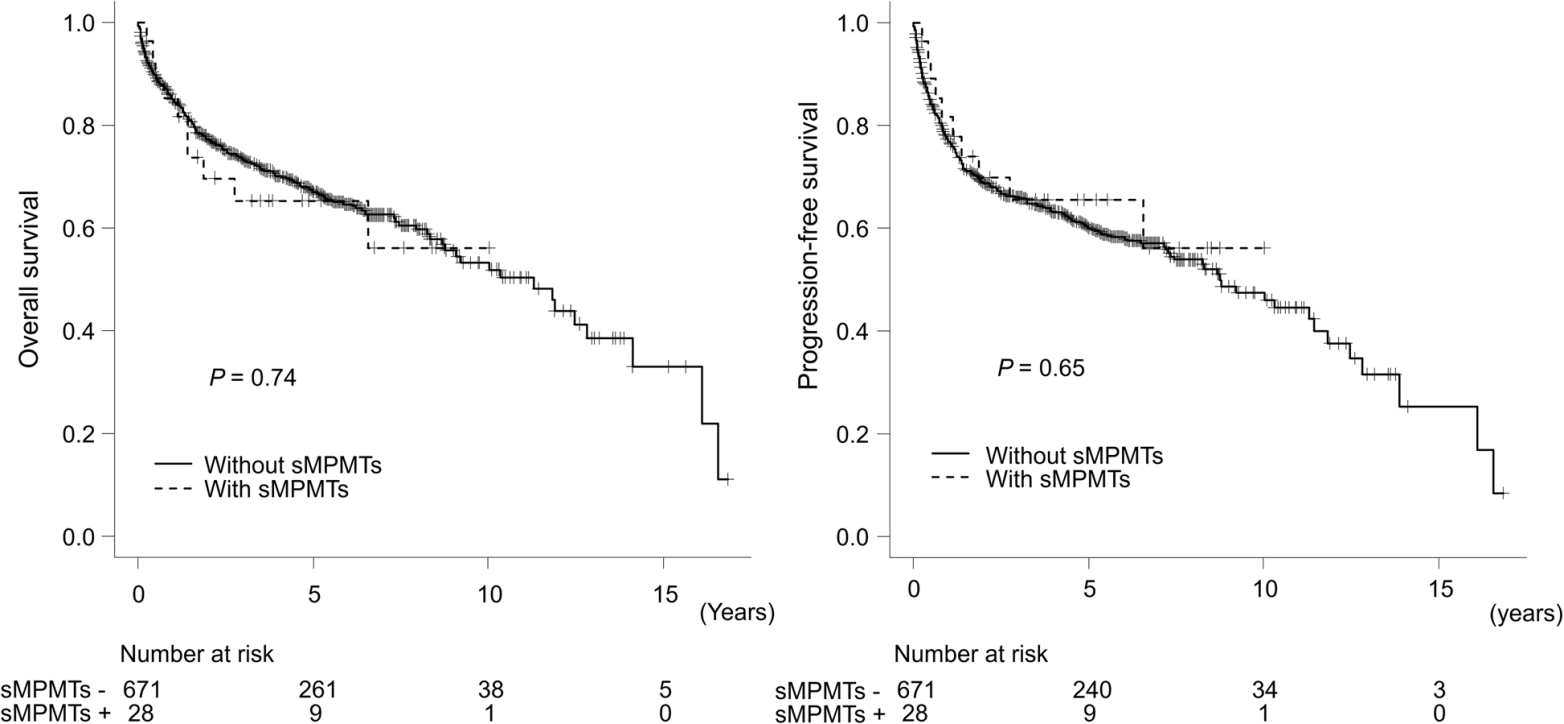
Kaplan-Meier curves of overall survival and progression-free survival stratified by the presence of sMPMTs in patients with DLBCL

**Fig. 4:**
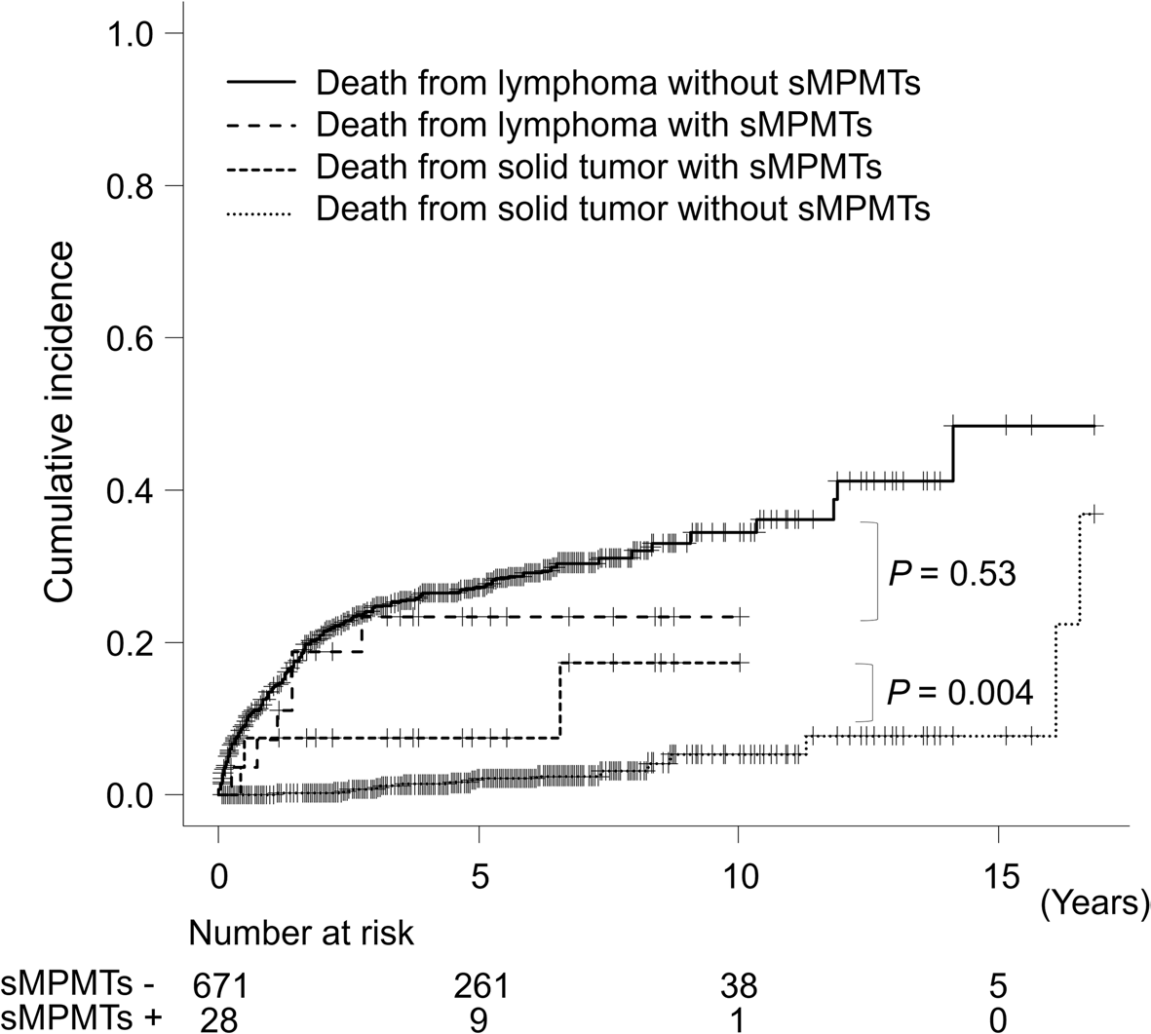
Cumulative incidence of death due to lymphoma or solid tumor stratified by the presence of sMPMTs in patients with DLBCL

No significant differences were observed in 5-year OS and PFS between patients with DLBCL with and without sMPMT (5-year OS: 65.4% and 66.9%, respectively; *P* = 0.74; 5-year PFS: 65.5% and 59.9%, respectively; *P* = 0.65) (Fig. 3). While the cumulative rate of death from lymphoma at 5 years was nearly the same in patients with and without sMPMT (23.4% and. 27.3%, respectively; *P* = 0.53), the 5-year cumulative rate of death from solid tumor in patients with sMPMT was significantly higher than those without sMPMT (7.4% vs. 2.1%, *P* = 0.004) (Fig. 4).

**Fig. 5:**
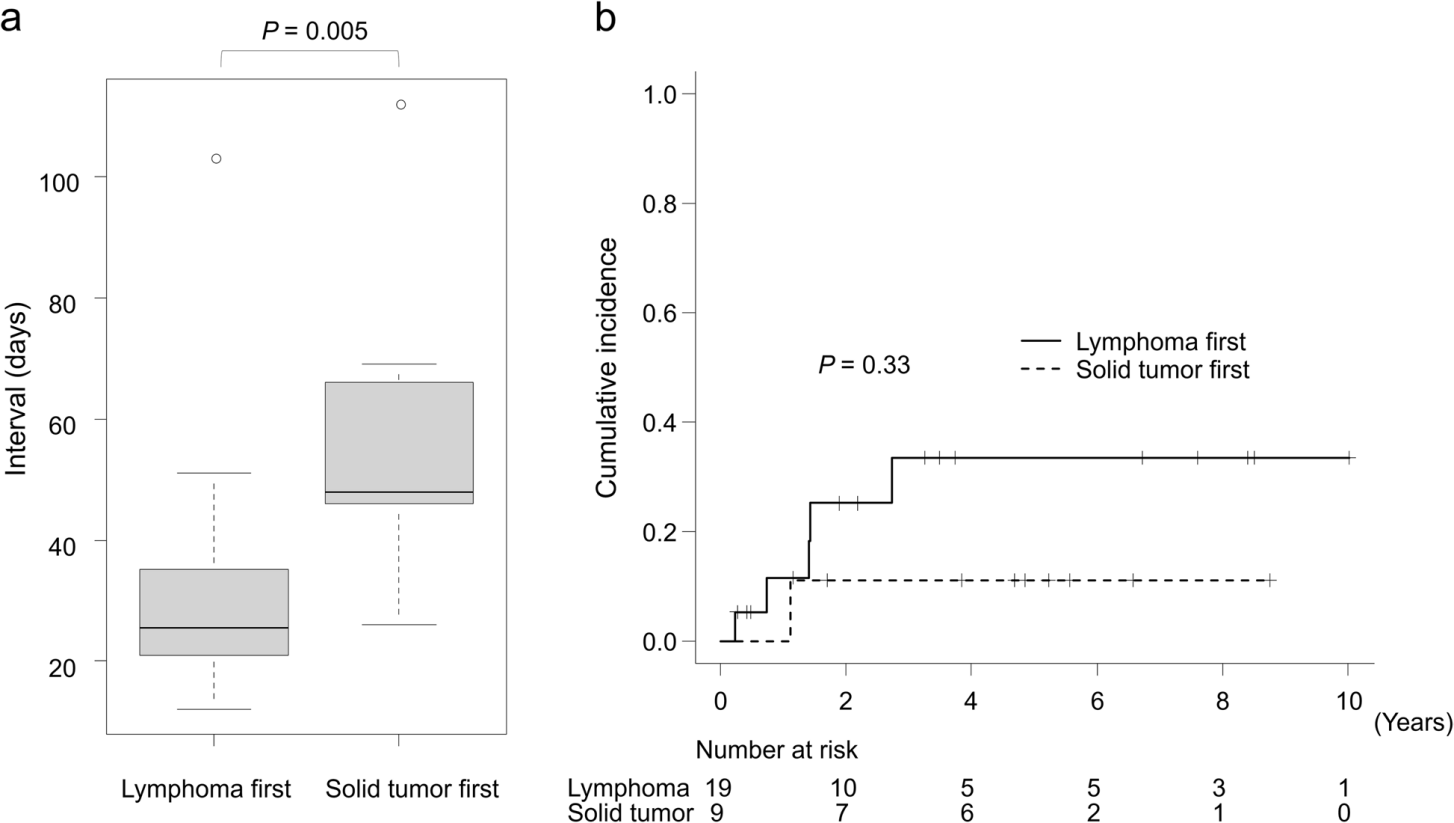
Interval between lymphoma diagnosis and chemotherapy commencement in terms of treatment sequence (**a**). Cumulative incidence of death due to lymphoma stratified by treatment sequence (**b**)

Comparison of the interval from the lymphoma diagnosis to chemotherapy commencement in the patients who received the lymphoma treatment first (*n* = 19) and those who received the solid tumor treatment first (*n* = 9) revealed a significantly longer interval in the latter group (median: 48 days and 25.5 days, respectively; *P* = 0.005) (Fig. 5a). However, the cumulative rate of death from lymphoma at 5 years tended to be higher in patients who received the lymphoma treatment first (30.2% and 11.1%, respectively; *P* = 0.33) (Fig. 5b).

**Fig. 6:**
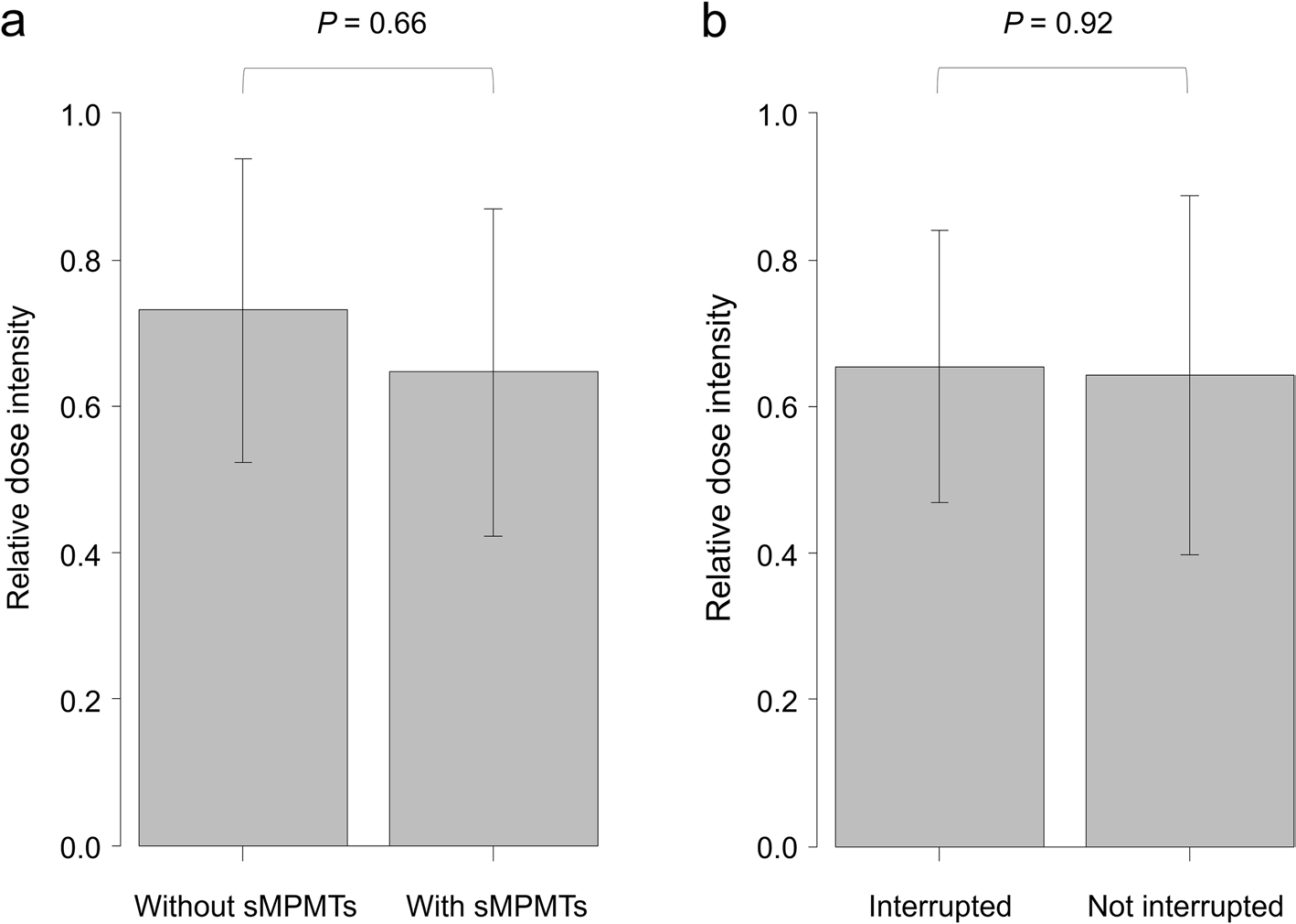
Relative dose intensity in terms of the presence of sMPMTs (**a**) and interruption of chemotherapy for lymphoma (**b**)

The RDI of R-CHOP and R-THP-COP tended to be lower in patients with sMPMT (64.7% and 73.0%, respectively; *P* = 0.066) (Fig. 6a). Investigation of the relationship between the RDI and interruption of chemotherapy for lymphoma due to therapy for solid tumor in patients with sMPMT revealed no significant difference between the group with (*n* = 7) and without (*n* = 15) interrupted chemotherapy (65.4% and 64.3%, respectively; *P* = 0.92) (Fig. 6b).

## Discussion

The present study demonstrated that, in indolent lymphoma patients, the prognosis of patients with sMPMTs was worse than those with sMPMTs. The mortality rate due to a solid tumor was significantly higher in patients with sMPMT regardless of tumor burden. The presence of sMPMTs did not significantly impact the survival outcomes of patients with DLBCL. However, the cumulative rate of death from solid tumors was significantly higher in patients with, than those without, sMPMT. The sequence of treatment did not significantly affect the outcomes or the RDI.

sMPMTs were observed in 5.8% (75 of 1285) of the lymphoma patients. In patients with DLBCL, sMPMTs occurred in 28 of 671 (4.1%) patients or roughly the same proportion as in previous reports [3, 4]. These figures may change depending on the examinations performed before treatment.

In patients with indolent lymphoma, those with sMPMTs had a worse 5-year OS rate than those without sMPMTs (73.4% vs. 87.8%, *P* = 0.028), and the cumulative rate of death due to a solid tumor was significantly higher in patients with sMPMT among those with high and low tumor burden (high tumor burden: 26.7% vs. 1.6%, *P* < 0.001; low tumor burden: 12.7% vs. 1.0%, *P* = 0.003). In most patients with low tumor burden indolent lymphoma, curative therapy for solid tumors was performed prior to chemotherapy for lymphoma. Considering together the low mortality rate from lymphoma, treating solid tumors first in patients with low tumor burden indolent lymphoma is considered acceptable. On the other hand, in high tumor burden indolent lymphoma, deaths due to lymphoma or solid tumors occurred at nearly an equal rate (31.9% vs. 26.7%). The decision as to which malignancy to treat first should be made individually. In patients with indolent lymphoma with sMPMTs, it is important to assess the status of each malignancy to determine whether early intervention is needed.

The baseline characteristics of the patients with a diagnosis of DLBCL with sMPMTs did not differ significantly from that of patients without sMPMTs, except in terms of sex and PS. The presence of sMPMTs did not affect 5-year OS or PFS, a finding that accords with the result of a previous study [3]. However, the cumulative mortality rate from solid tumors was significantly higher in patients with sMPMT.

As might be expected, the interval from diagnosis to lymphoma treatment was significantly longer in patients who received treatment for their solid tumor first. Delayed treatment of lymphoma is reportedly associated with worsening prognosis [9–11]. However, in the present study, the cumulative rate of death due to lymphoma tended to be higher in patients who received the lymphoma treatment first possibly because the patients with poor-risk DLBCL were more likely to be treated early. In fact, patients with B symptoms, higher LDH, and a higher risk of IPI were more common in the group who received lymphoma treatment first although the difference was not significant. Delaying treatment in patients with DLBCL with early-stage disease or low LDH is apparently not associated with poor outcomes [11]. Therefore, treating solid tumors and delaying lymphoma treatment is considered acceptable in patients with low-risk lymphoma while chemotherapy should be started as early as possible for high-risk lymphoma.

The RDI of R-CHOP and R-THP-COP tended to be lower in patients with sMPMTs not because of the interruption of chemotherapy to treat the solid tumors but because patients who were elderly or had a generally low RDI were more common in the group with sMPMTs (27.3% and 13.3%, respectively). Given that interruption of chemotherapy did not lower the RDI, treatment for solid tumors may begin after or during chemotherapy for DLBCL; the order of treatment depends on the status of each malignancy per individual.

The present study had some limitations; it was a retrospective, non-randomized study enrolling a small cohort. In particular, a treatment bias was introduced by the physicians making treatment decisions at their own discretion. However, to the best of our knowledge, the present study is the largest to investigate the impact of sMPMTs on patients with newly diagnosed malignant lymphoma. Considering the rarity of sMPMTs in patients with lymphoma, prospective studies may be difficult to conduct. We believe that this study can provide some useful insights into the management of these patients.

## Conclusions

In conclusion, in patients with indolent lymphoma, those with sMPMTs had a significantly worse prognosis, which was considered to be due to the high mortality rate from solid tumors. Treatment of solid tumors may be prioritized if the lymphoma burden is low. The presence of sMPMTs was not a significant prognostic factor in patients with the diagnosis of DLBCL. The status of each malignancy needs to be assessed individually in patients with sMPMTs to determine the need for early treatment.

## Data Availability

The datasets used and/or analyzed during the current study are available from the corresponding author on reasonable request.

## Abbreviations

sMPMTs: Synchronous multiple primary malignant tumors
DLBCL: Diffuse large B-cell lymphoma
OS: Overall survival
PFS: Progression-free survival
WHO: World Health Organization
PS: Performance status
ECOG: Eastern Cooperative Oncology Group
IPI: International Prognostic Index
NCCN: National Comprehensive Cancer Network
LDH: Lactate dehydrogenase
GELF: Groupe d’Etude des Lymphomes Folliculaires
CT: Computed tomography
PET: Positron emission tomography
R-CHOP: Rituximab, cyclophosphamide, doxorubicin, vincristine, and prednisone
THP: Tetrahydropyranyl adriamycin
RDI: Relative dose intensity
